# Heart rate responses in critical care trainees during airway intubation: a comparison between the simulated and clinical environments

**DOI:** 10.1186/s12873-023-00832-8

**Published:** 2023-06-10

**Authors:** Jackson Ji, Bridget Langley, Rachel Zordan, Julian van Dijk, Heidi Helene Graham Thies, Anjalee Brahmbhatt, Clarissa Torcasio, Neil Cunningham

**Affiliations:** 1 Melbourne Medical School, Faculty of Medicine, Dentistry and Health Sciences, University of Melbourne, Melbourne, Australia; 2 Department of Anaesthesia and Acute Pain Medicine, St Vincent’s Hospital, Melbourne, Australia; 3 Education and Learning, St Vincent’s Hospital Melbourne, Melbourne, Australia; 4 Department of Emergency Medicine, St Vincent’s Hospital, Melbourne, Australia

**Keywords:** Medical simulation, Airway intubation, Critical care doctors, Heart rate, Stress response

## Abstract

**Objective:**

This study aimed to compare the heart rate response to stress during airway intubations in clinical practice and a simulated environment.

**Methods:**

Twenty-five critical care registrars participated in the study over a 3-month period. Heart rate data during intubations was recorded by a FitBit® Charge 2 worn by each participant during their clinical practice, and during a single simulated airway management scenario. The heart rate range was calculated by subtracting the baseline working heart rate (BWHR) from the maximum functional heart rate (MFHR). For each airway intubation performed participants recorded an airway diary entry. Data from intubations performed in the clinical environment was compared to data from a simulated environment. Heart rate changes were observed in two ways: percentage rise (median) across the 20-min intubation period and; percentage rise at point of intubation (median).

**Results:**

Eighteen critical care registrars completed the study, mean age 31.8 years (*SD* = 2.015, *95% CI* = 30.85–32.71). Throughout the 20-min peri-intubation recording period there was no significant difference in the median change in heart rates between the clinical (14.72%) and simulation (15.96%) environment (*p* = 0.149). At the point of intubation there was no significant difference in the median change in heart rate between the clinical (16.03%) and the simulation (25.65%) environment groups (*p* = 0.054).

**Conclusion:**

In this small population of critical care trainees, a simulation scenario induced a comparable heart rate response to the clinical environment during intubation. This provides evidence that simulation scenarios are able to induce a comparable physiological stress response to the clinical environment and thus facilitates effective teaching of a high-risk procedure in a safe manner.

**Supplementary Information:**

The online version contains supplementary material available at 10.1186/s12873-023-00832-8.

## Introduction

**Fig. 1:**
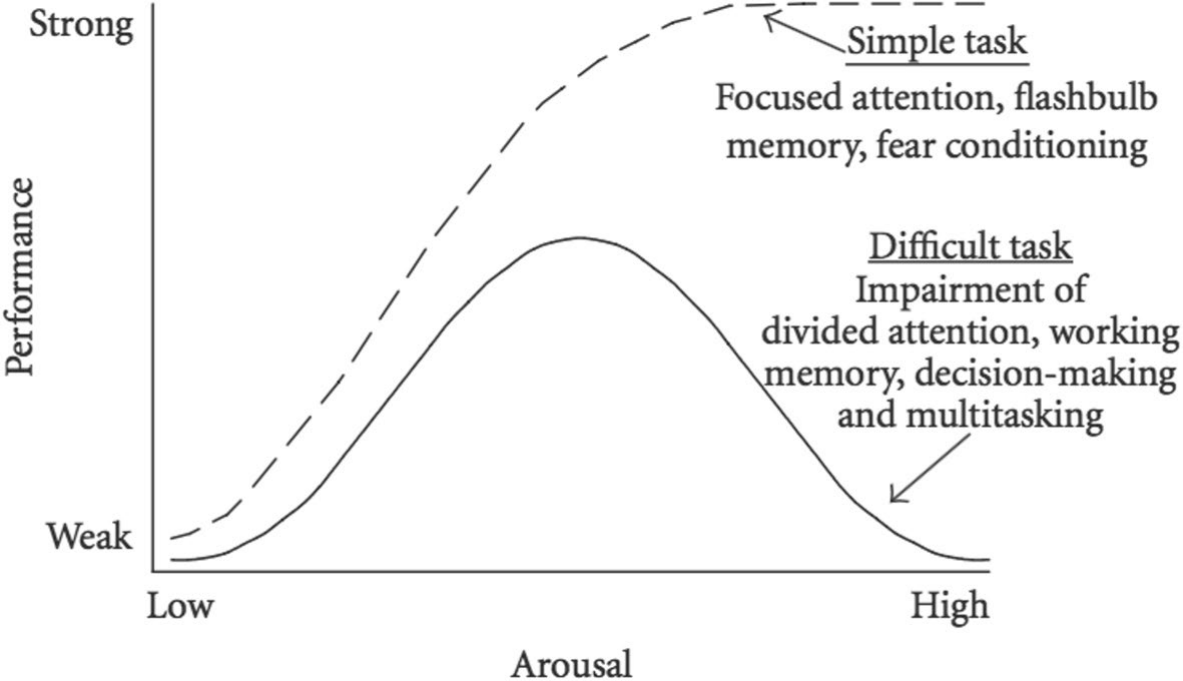
The Yerkes-Dodson Law

Medical practitioners are regularly exposed to high pressure situations in their clinical practice which can result in considerable stress. Stress is a set of adaptive responses to environmental demands [1, 2], and is triggered when the perceived demands outweigh the individual’s resources [3]. High levels of stress have been shown to impair performance [3], although a degree of stress correlates with peak performance. This relationship between stress and performance of complex tasks is described by the Yerkes-Dodson law [4, 5] (see Fig. 1).

Changes in heart rate, blood pressure, and cortisol levels have been used to measure the physiological manifestations of stress [1, 6–11]. Tachycardia is a recognised sign of stress due to increased sympathetic nervous activity and is relatively simple to measure, thus heart rate is often used to measure stress response [1, 10]. The stressors encountered by medical practitioners include those related to a patient’s physical and psychological state, the task or procedure to be undertaken, and those pertaining to the individual practitioner and their team. While the nature of stress is complex, it is feasible that reducing the stress response of medical practitioners may improve clinical performance via reduced errors.

Students and junior medical staff are rarely exposed to unstable patients requiring a critical procedure, such as airway intubation. Simulation-based education primarily aims to replicate these scenarios in a safe environment, where participants are tested on multiple levels (cognitive, procedural, and affective) without the fear or repercussions of harming a patient [12]. Simulation allows participants to be exposed to a wide variety of problems and work conditions, including common scenarios that they would be expected to manage, unusual cases that they may rarely encounter, and critical scenarios that would normally be managed by a more senior person. This assists with the acquisition of psychomotor skills (e.g. procedural steps), behavioural skills (e.g. communication and leadership), and cognitive skills (e.g. decision making). Simulation-based education can be tailored to an appropriate level for the participants’ experience and learning outcomes. In addition to skill development, the ability for real time assessment and immediate feedback has been shown to have the greatest potential for retention of knowledge, resulting in improved performance [13]. This has been adapted into a ‘scaffolding’ type of supervision, where the amount of guidance from the educator decreases as the trainee becomes more proficient [12].

While simulation is widely used, its ability to evoke similar stress responses to the clinical environment has not been validated. Only two studies have compared the stress levels in simulated scenarios with the clinical environment. However, they have produced conflicting results. Dias and Neto (2016) [8] found that simulation may evoke similar acute stress responses in comparison to the emergency environment, whereas Baker et al. (2017) [14] found a significantly lower stress response induced by simulation than the clinical environment.

This study aims to determine whether heart rate response to stress during airway intubations is the same in clinical practice as in a simulated environment among critical care trainees (anaesthetics, emergency medicine (EM) and intensive care unit (ICU) registrars).

## Method

### Study design

This was a prospective observational study, conducted at a tertiary hospital over a 3-month period.

### Participants

All registered trainees in critical care specialties (anaesthetics, EM, and ICU) were eligible to participate and there were no exclusion criteria. Informed consent was sought and obtained. Participants were assigned a study number in order of recruitment to blind the supervising investigators.

### Procedure

Each participant completed i) a demographic questionnaire and ii) two psychological measures, and iii) a treadmill exercise test according to a modified Bruce Protocol [15] to determine maximal heart rate. Heart rate data was recorded using a heart rate monitor worn during clinical duties for a 3-month period. Participants completed an airway intubation diary for each intubation performed. Finally, participants attended a simulation session in which they were required to manage an endotracheal intubation.

## Measures

### Demographic questionnaire

Prior to participation in the study, each participant completed a questionnaire including: demographic characteristics including age, clinical role in the hospital (anaesthetics, ICU or ED), experience in that role (months), level of training, subjective level of expertise in intubation, and number of endotracheal intubations.

### Psychological assessment tools

The DASS-42 is a self-reported scale designed to assess stress, anxiety and depression. It is a 42-item scale on which participants’ rate to what extent they agree with each statement. Each statement is associated with one of the above psycho-emotional states, with agreement ranging from *‘does not apply to me at all*’ (0) to *‘applies to me very much’* (3). A higher score indicates a higher level of that particular state [16].

The STAI-AD was used to measure baseline anxiety associated with a subjective stress response. It consists of a ‘state anxiety’ and a ‘trait anxiety’ scale used to measure the anxiety associated with the participant’s experience of stress. The STAI-AD consists of 20-items for each ‘state’ and ‘trait’ anxiety, with a total of 40 items (e.g. *‘I am tense’*). Participants rate their level of agreement using a 4-point scale to each statement, regarding how they felt at that given moment and in general. Scores on the STAI-AD range from 20 to 80 with a higher score indicating greater anxiety. The STAI-AD was administered prior to participation in the study as a measure of participants’ baseline stress response, as anxiety is an emotional state strongly associated with the stress response [17].

### Treadmill exercise test

Maximum functional heart rate (MFHR) was calculated for each participant using a treadmill exercise test according to a modified Bruce protocol [15] (see Appendix 1). Participants ran at each stage for 3 min before the speed and gradient was increased to progress to the next stage. MFHR was recorded and the test concluded at the point at which the participant was unable to continue, or when they had completed all levels of the modified Bruce protocol. For participants unable or unwilling to participate in the stress test, the MFHR was calculated at their age in years subtracted from 220 [18].

### Heart rate measurement

Heart rate was continuously measured using a FitBit® Charge 2 (FitBit Inc 2007, California, United States) and recorded throughout the participant’s work day. Second-by-second heart rate data was extracted for analysis from 15 min prior to the recorded intubation time to 5 min post recorded intubation time. This 20 min intubation time period was selected due to the nature of the study population and expected intubation events. It was anticipated that the majority of intubations would be elective procedures completed by anaesthetic trainees, with a lesser amount performed as emergency procedures in one of the three critical care environments. We estimated that preparation time for elective intubation would take approximately 15 min, therefore capturing any anticipatory heart rate changes. Any stress-inducing post-intubation events such as desaturation, misplacement and re-insertion of the tube, as well as recovery from the complex task induced stress response, were intended to be captured in the 5 min following intubation. Intubation time was based on the online diary entries made by participants.

Heart rate changes were observed in two ways: percentage rise (median) across the 20-min intubation period, and percentage rise at point of intubation. The heart rate range was calculated by subtracting the baseline working heart rate (BWHR) from the maximum functional heart rate (MFHR). The BWHR was calculated by generating the median of the minimum heart rate from each intubation that the participant recorded from the clinical environment. The BWHR was selected as trainees were only expected to wear the FitBit® when they were in the workplace. The heart rate change was selected as our comparison data over absolute heart rate values as we anticipated variability in the heart rate ranges within individuals in the study population. Clinical heart rate changes were collected as a median for all clinical intubation data entries, the resultant number then compared with the heart rate changes from the simulation scenario.

### Clinical airway intubation diary

Participants completed an online diary for each intubation. Data collected included: date and time of intubation, type of intubation, emergency case (yes/no), subjective case difficulty, perceived stress level, level of supervision, and the Samn-Perelli 7-point fatigue scale (1 = fully alert, wide awake, 7 = completely exhausted, unable to function effectively). As studies have shown that caffeine can affect heart rate [19], participants’ daily consumption of caffeine (tea, coffee or other caffeinated drinks) were recorded. Any regular medication was also noted.

### Simulated airway scenario

Participants attended a simulated scenario requiring the trainee to perform an airway intubation. This took place in an artificial environment with a high-fidelity mannequin representing a deteriorating patient. The session was tailored to the participant’s current training environment (Emergency Department, Intensive Care Unit, Anaesthetic Department). This allowed the scenarios to maintain consistency in timing of phases of clinical deterioration, diagnostic difficulty, and management difficulty. We did not attempt to adjust scenario difficulty for each individual’s perceived level of expertise as the trainees ranged from junior registrars to senior fellows and this would have introduced additional variability into the scenario. Rather, the scenario was designed to be a replicable emergency situation expected to be to manageable for all participants but difficult enough to challenge them. The simulation concluded one-minute post intubation, or after fifteen minutes if the intubation was not successful. See Appendix 2 for a description of the simulation scenario.

### Data management and statistical analysis

As there were no comparable studies at the time which addressed the heart rate response to stress during airway intubation when comparing simulation environment with the clinical environment, sample size was determined for this study by participant availability. All data were analysed with SPSS software V23.0 (SPSS Inc. Chicago, IL), and graphed with GraphPad Prism version 5.00 for Mac (GraphPad Software, La Jolla, CA).

Demographic data were described using mean and standard deviation and heart rate data were described using median and interquartile range when appropriate. Median heart rate changes were selected given the small sample size, to reduce the power of outliers in the analysis.

Prior to all analyses and given the sample size of less than 50, a univariate normal distribution was tested by the Shapiro–Wilk test for the physiological responses to airway intubation. The level of statistical significance was set at *p* < 0.05.

Grouped data were analysed using a Wilcoxon signed rank test for comparison of median changes in heart rate over the 20-min peri-intubation recording period between the clinical and simulation environment. Mann–Whitney U test was applied for comparison of median changes in heart rate at point of intubation between the clinical and simulated environment.

Individual data were analysed using Wilcoxon signed rank test was used to compare the heart rate changes at the time of intubation for individual study participants between the clinical and simulation environments.

## Results

Between February and June 2017, 25 trainees agreed to participate. One participant withdrew, five were excluded due to no clinical intubation data and one was excluded due to no simulation intubation data, leaving 18 participants in the analysis.

### Demographic results

Participants had a mean age of 32 years (*SD* = 2.015, *95% CI* = 30.85–32.71). Further demographic data are summarised in Table 1.

**Table 1:** Characteristics of study participants (*n* = 18)

	**Number**	**%**	**95% CI**
**Gender**			
Male	9	50	0.27–0.73
Female	9	50	0.27–0.73
**Months of Experience**			
1 to 12	4	22.2	0.03–0.41
13 to 24	5	27.8	0.07–0.49
25 to 36	1	5.6	0.00–0.16
37 to 48	5	27.8	0.07–0.49
49 to 60	3	16.7	0.00–0.34
**Training discipline**			
Anaesthetics	10	55.6	0.33–0.79
Emergency	4	22.2	0.03–0.41
Intensive Care	4	22.2	0.03–0.41
**Subjective Expertise in intubation**			
1 – Novice	1	5.6	0.00–0.16
2	1	5.6	0.00–0.16
3	7	38.9	0.16–0.61
4 +-Expert	9	50.0	0.27–0.73
**Number of intubations performed**			
1 to 100	5	27.8	0.07–0.49
101 to 1000	9	50.0	0.27–0.73
1000 +	4	22.2	0.03–0.41
	**Mean**	**SD**	**95% CI**
**Psychological Measures**			
DASS-Depression	4.06	4.19	2.23—5.13
DASS-Anxiety	3.39	4.68	1.69—5.35
DASS-Stress	8.83	6.59	6.61—11.31
STAI-anxiety	35.22	10.53	30.36–40.08
STAI-trait	37.03	8.92	32.91–41.15

The results of the psychological measures (DASS-42, STAI-AD) are included in Table 1. Results on both measures were comparable to normative data for a non-clinical adult population [17, 20]**.**

Participant mean caffeine intake on a daily basis was 1.94 beverages (*SD* = 0.54, range = 1–5). Two participants were taking medications known to affect cardiac function. Given the small number of participants reporting medication use and the low levels of caffeine consumed, no adjustments were made for this.

The 18 participants completed 202 intubations (range = 1–47). Eleven of the intubations did not have corresponding heart rate data recorded due to technical issues with the FitBit® (range = 1–47). The remaining 191 intubation entries were analysed against the 18 simulation scenarios, with participant-specific data represented in Table 2.

**Table 2:** Participant physiological and intubation data

Participant Training Discipline	Number of clinical intubations (missing HR data)	Clinical Environment median HR change, %	Simulation Environment HR change, %	Baseline working HR, bpm	Maximum HR, bpm
Anaesthetics	24 (1)	21.65	29.00	82.5	198
ICU	5	26.98	58.73	73	136
ED	2 (1)	23.97	12.40	79.5	140
ICU	2	24.55	26.36	59	169
ED	5 (1)	16.96	29.46	75	187
Anaesthetics	11	14.44	16.67	74	164
ICU	47 (4)	20.24	13.10	70	154
Anaesthetics	21	17.54	19.30	59	173
Anaesthetics	6 (1)	14.62	27.69	80	145
ICU	1	22.89	14.46	77	160
Anaesthetics	1	21.11	30.89	68	191
Anaesthetics	8	18.06	31.48	77	185
Anaesthetics	6	14.10	52.56	71	149
Anaesthetics	28 (3)	19.05	30.16	55.5	150
Anaesthetics	8	7.79	9.83	60	182
ED	25	16.96	14.29	62	174
ED	1	46.15	46.15	94	159
Anaesthetics	1	10.87	13.04	58	150

### Comparison of heart rate response to stress (clinical vs simulation)

#### Over the intubation period

**Fig. 2:**
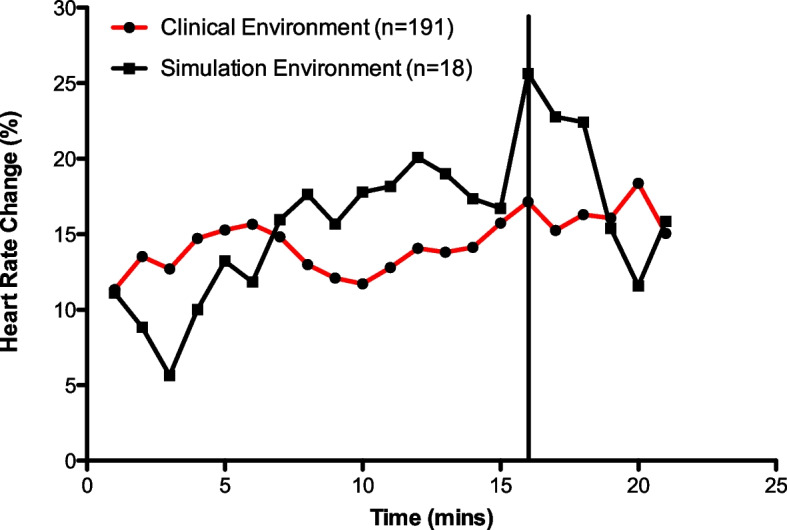
Comparison of HR changes during intubation period

The participants exhibited a statistically significant increase in heart rate over the recorded period in all time points in both environments as shown in Fig. 2. A Wilcoxon signed-rank test showed that the percentage change in median heart rates were not statistically different between the clinical environment (14.72 *IQR* = 12.89–15.70) and simulation environment (15.96 *IQR* = 11.71–18.59) (*p* = 0.149) across the group.

#### At the point of intubation

**Fig. 3:**
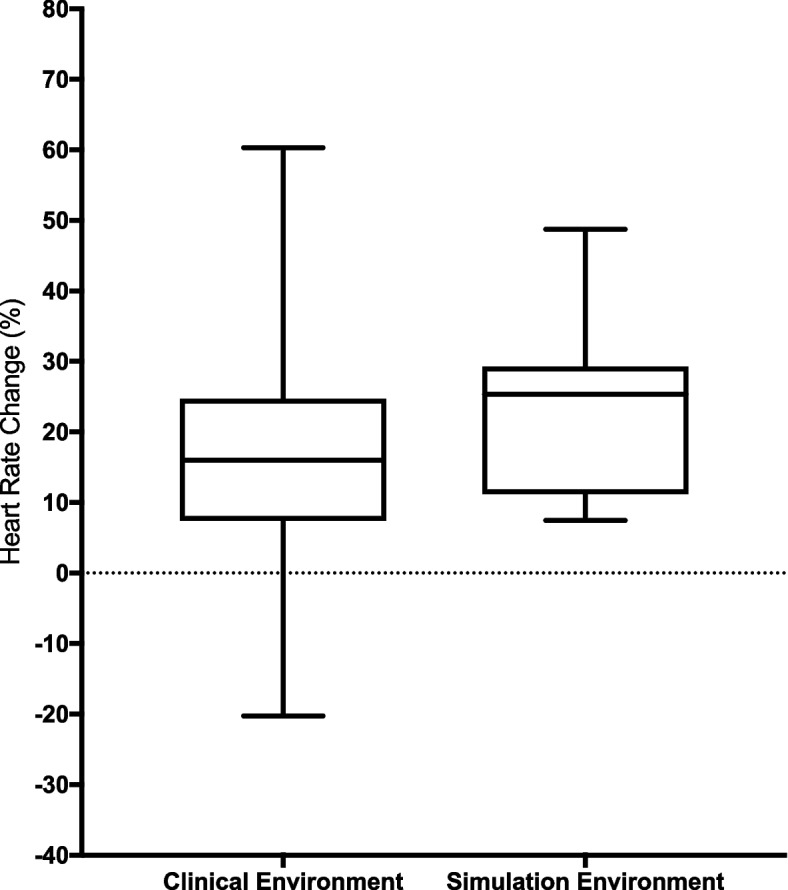
HR changes at the point of intubation

A Mann–Whitney U test was conducted to determine if there were differences in heart rate changes between the clinical (*n* = 191) and simulation environments (*n* = 18) at the time of intubation (t = 16) as shown in Fig. 3. Distributions of the heart rate changes were similar, as assessed by visual inspection. Median heart rate changes were not statistically significantly different between the clinical (16.03%) and simulation environments (25.65%) across the group, *U* = 1245, *z* = -1.933, *p* = 0.054.

### Individually matched stress response between the two environments at intubation

**Fig. 4:**
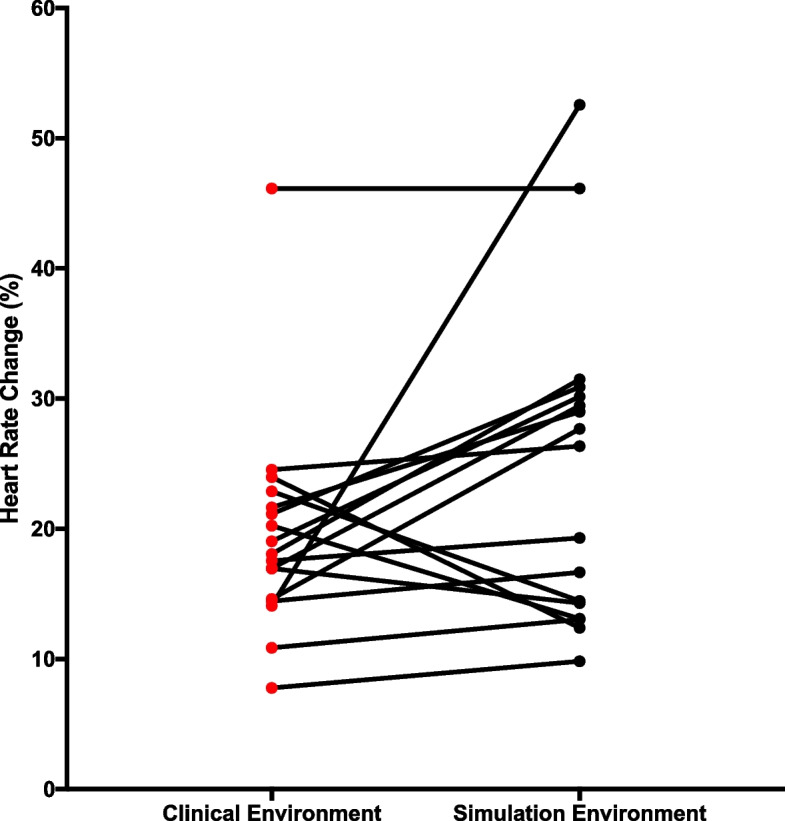
Individually matched comparison

Eighteen participants had heart rate data recorded to understand whether the physiological response of intubations could be compared between the clinical and simulation environments. Exploration of the data found an extreme outlier (simulation heart rate change = 58.7%), which was removed from subsequent analysis. Of the remaining 17 participants: simulation elicited a larger increase in heart rate changes in 12 participants; the clinical environment elicited a larger increase in heart rate changes in 4 participants; and one participant had no change from clinical to simulation environment (Fig. 4). A Wilcoxon signed-rank test determined that there was no significant median increase in heart rate change among the participants at intubation in simulation (24.5%) compared to clinical environment (19.4%), *z* = -1.758, *p* = 0.079.

## Discussion

Our hypothesis was that the heart rate changes from airway intubation would be similar between clinical and simulation environments in critical care trainees. We observed real-time dynamic changes in heart rate in both environments. There was no significant difference in the median changes of heart rates between each environment across the entire peri-intubation recorded period or at the point of intubation within this study population.

The temporal characteristics of the heart rate changes demonstrated some unanticipated findings. In the clinical environment, participants demonstrated an anticipatory increase in heart rate, which peaked ten minutes prior to the recorded intubation time. This could be due to anxiety related to performing a complex task, or arousal linked to pre-emptive planning for failure or success. A similar anticipatory stress response has been reported in doctors prior to breaking bad news [7, 21].

As expected, a second peak in heart rate was observed at the point of intubation. This reflects a similar heart rate trajectory reported by Baker et al. while examining heart rates during rapid sequence induction [14]. Interestingly, a third peak in heart rate change occurred four minutes post intubation in some participants. This could be attributed to either a patient or work-related stress, such as decreased oxygen saturation or the transfer of the patient, or a delayed stress response during reflection upon a difficult task.

In comparison, the simulated temporal characteristics demonstrated an extended period of anticipatory heart rate response. This could be contextual anticipation due to participants expecting that a negative outcome would occur in the simulation scenario, due to the prior exposure to simulated sessions. Similar to the clinical environment, the point of intubation demonstrated the largest change in heart rate.

When comparing the median heart rate changes at the time of intubation induced in each environment, there was no statistically significant difference. This suggests that heart rate changes during intubation can be replicated in the simulation environment. This result is consistent with previous studies that have found acute stress responses were similar to real emergency situations [8].

The individually matched comparison of participants’ median heart rate changes at the time of intubation did not show a statistically significant difference between the two environments. This differs to the findings of Baker et al., who compared the stress response of anaesthetic trainees when conducting rapid sequence induction [14]. Their study simulation was unable to replicate the stress of the technical procedure and to our knowledge it is the only other study to directly compare stress of simulation with the clinical environment.

Two participants in this study showed a marked increase in heart rate change during simulation. This may be the results of previous simulation experience, either too much (negative) or too little (naïve and fearful), or other confounders that exaggerated the stress response that day. The experience of the participants may also be a factor in the variability of both arousal and performance. Participants reported a range of experience, from novice to advanced trainee, and it is possible that for some of the more inexperienced participants, the simulated intubation was interpreted as complex by the novices, and simple by the advanced participants. As the Yerkes-Dodson Law demonstrates [4], the stress-performance effect is impacted by the complexity of the task, the perceived complexity for the individual in this setting would depend upon experience level. However, despite the small study population, the results across the group suggest that the simulation scenario was an appropriate intervention.

### Limitations

There are several limitations in this study. Firstly, similar studies on stress induced during simulation have looked at other markers of physiological stress such as blood pressure, cortisol levels, and skin conductance [1, 3, 6–11, 22–25]. The inability to analyse these factors in the clinical environment stemmed from the unpredictable nature associated with the timing of intubations and patient safety factors. The use of wrist worn heart rate monitors has been shown to be less accurate than gold-standard ECG monitoring, with the FitBit® Charge 2 showing an under-estimating bias in a small study [26]. Each FitBit® Charge 2 was purchased new and only used by each individual for the purposes of the study, they were not further calibrated against gold-standard ECG monitoring. The large amount of data collected in the working environment in this study was able to show consistent changes in heart rate among participants despite the reduction in precision. Precision ECG monitoring would not have been possible to perform in the working environment, which would have prohibitively limited the scope of the study.

Secondly, the majority of the clinical data was recorded by anaesthetic trainees when conducting elective surgery intubations. Comparing the heart rate changes in this setting with a high-fidelity emergency simulation scenario does not completely replicate the clinical situation in which the participants recorded their data. This may have resulted in procedural bias. To reduce confusion of data in future studies, a comparison of elective clinical intubation data with an elective simulation scenario would be appropriate. The simulation scenario concluded at one-minute post intubation with the intent of reducing the risk of participants losing the psychological fidelity of the scenario post-intubation. It may be preferable to continue the scenario with post-intubation tasks, rather than the potentially confounding effects on heart rate of early debriefing following the simulation. This would also allow a closer analysis of the third peak noted at the 4-min post intubation point in some individuals.

Thirdly, the sample size of the study was small. There was a population of 25 critical care trainees with only 18 able to provide full data which may limit the external validity of the study for other settings. This small sample size contributed to greater variations in the simulation heart rate data compared to the clinical heart rate data. However, statistical analyses applied were sensitive to this. The outlier was removed prior to statistical analysis as their data was extreme and may have skewed results bearing in mind the small sample size. This outlier data would not have changed the results from being consistent with the null hypothesis.

Finally, no adjustments were made for caffeine intake or for medications that affect cardiac function. Caffeine was recorded as daily intake and was consistent for individuals across the two test environments. The precise timing of caffeine dosing could have affected the pre-intubation HR at an individual level for specific intubations, but this was not recorded. For the two participants who were taking medications known to affect cardiac function, intake was consistent across the two test environments. The potentially attenuating affect on HR changes were the same for those individuals in their treadmill exercise test (MFHR), simulated and clinical environments.

The strengths of the study included compliance among participants with wearable technology for a prolonged working period, reducing the risk of a Hawthorn effect upon the clinical data. Only 5.4% of all intubations were lost to technological (partially recorded data) or compliance issues (participants forgetting to wear their FitBit®).

Further research could look at whether repeated exposure to different simulation scenarios of varying difficulty attenuates the induced stress response. The ability to demonstrate similar heart rate changes in the simulated environment could lead to further analysis of the efficacy of specific stress attenuation training for individuals.

## Conclusions

In this small population of critical care trainees, a simulation scenario induced a comparable heart rate response to the clinical environment during intubation. This provides evidence that simulation scenarios are able to induce a comparable physiological stress response to the clinical environment and thus facilitates effective teaching of a high risk procedure in a safe manner.

## Supplementary Information

**Additional file 1.**

**Additional file 2.**

## Data Availability

The datasets used and/or analysed during the current study are available from the corresponding author on reasonable request.

## Abbreviations

EM: Emergency medicine
ICU: Intensive care unit
BWHR: Baseline working heart rate
MFHR: Maximum functional heart rate
DASS-42: Depression, anxiety and stress scale, a 42 point self-report scale
STAI-AD: State trait anxiety inventory for adults
