# Supplementary material for: Heart rate responses in critical care trainees during airway intubation: a comparison between the simulated and clinical environments
Source: BMC Emerg Med. 2023 Jun 10;23:66. doi: 10.1186/s12873-023-00832-8 (PMC10257286; doi:10.1186/s12873-023-00832-8)
Supplement: Supplementary file 1 — Additional file 1. [file 12873_2023_832_MOESM1_ESM.pdf]

Modified Bruce Protocol*

**Stage**	**Time (minutes)**	**Km/hour**	**%Gradient**
1	3	2.7	10
2	3	4.0	12
3	3	5.4	14
4	3	6.9	15
5	3	8.7	15
6	3	10	15
7	3	11.5	15

*Modification Calculation

$$mets = \frac{(speed * 0.1) + (speed * 1.8 * grade) + 3.5}{3.5}$$
