# Supplementary material for: Heart rate responses in critical care trainees during airway intubation: a comparison between the simulated and clinical environments
Source: BMC Emerg Med. 2023 Jun 10;23:66. doi: 10.1186/s12873-023-00832-8 (PMC10257286; doi:10.1186/s12873-023-00832-8)
Supplement: Supplementary file 2 — Additional file 2. [file 12873_2023_832_MOESM2_ESM.pdf]

The environment for the simulated airway scenarios was adapted to be in an area in which the trainee usually worked. As such, the setting was either the high dependency unit (HDU), post-anaesthesia care unit (PACU) or the emergency department (ED). A confederate played the role of a nurse.

The simulated patient developed angioedema and urticaria following the administration of morphine for analgesia, reported difficulty breathing and became progressively hypoxic due to airway obstruction.

Instructions provided to the participants before they entered the room: **"You have been asked to review a patient in ED/ICU/PACU"**

**Phase**	**Clinical Features**	**Confederate**
Start<br>Difficulty Breathing	Alert/Anxious<br>Swollen Tongue<br>Stridor<br>Wheeze<br>Respiratory Rate 34<br>Truncal Rash<br>SpO2 91-92% (on O2)<br>HR 94 sinus<br>SBP 135 – 140 mmHg	"I am concerned he may be having an allergic reaction"<br><br>"I gave the morphine 5 minutes ago."<br><br>Patient Voice – Stridor only no words spoken
At time 2 minutes	SpO2 86-88%<br>HR 110 sinus<br>SBP 145mmHg	"He is looking worse"<br>"I am concerned that he is having more trouble breathing"<br><br>Patient Voice – Stridor only no words spoken
At time 6 minutes	SpO2 82-84%<br>Fluctuating conscious state with eyes opening /closing<br>HR 110 with frequent VEs	
At time 8 minutes	SpO2 78%	
Manually<br>Selected<br>Intubation ETT<br>This may occur at any time point in the scenario	Unconscious<br>100% paralysis<br>SpO2 92%<br>HR 75 sinus<br>SBP 165 mmHg (will depend on medication used)	
